# Effect of Natural Compounds on NK Cell Activation

**DOI:** 10.1155/2018/4868417

**Published:** 2018-12-25

**Authors:** Malgorzata Grudzien, Andrzej Rapak

**Affiliations:** Laboratory of Tumor Molecular Immunobiology, Ludwik Hirszfeld Institute of Immunology and Experimental Therapy, Wroclaw 53-114, Poland

## Abstract

Natural killer (NK) cells are lymphocytes of the innate immune system that survey the body for stressed and abnormal cells. The integration of signals that they receive through various inhibitory and activating cell surface receptors controls their activation and ability to kill target cells and produce cytokines. In this manner, phenotypically and functionally distinct subsets of NK cells help protect against microbial infections and cancer and shape the adaptive immune response. NK cells can use two different mechanisms to kill their targets, either by cytotoxic granule exocytosis or by induction of death receptor-mediated apoptosis. Death ligands belong to the tumor necrosis factor (TNF) family of ligands. Upon release in close proximity to a cell slated for killing, perforin forms pores in the cell membrane of the target cell through which granzymes and associated molecules can enter and induce apoptosis. NK cells are also involved in antibody-dependent cellular toxicity via the CD16 receptor. In addition to target recognition, NK cells can be also activated by treatment with multiple compounds with stimulatory properties. Apart from interleukins, which belong to the best characterized group of NK cell-stimulating compounds, vitamins and constituents extracted from plants also display the ability to activate NK cells. The current review characterizes several groups of NK cell-activating compounds: vitamins belonging to classes A, B, C, D, and E, polysaccharides, lectins, and a number of phytochemicals used in cancer research, exhibiting stimulatory properties when applied to NK cells. Although in most cases the exact mechanism of action is not known, constituents described in this review seem to be promising candidates for NK cell-stimulating drugs.

## 1. Introduction

Natural killer (NK) cells have been identified in the early 1970s due to a series of experiments regarding cytotoxicity in cancer patients [1]. Phenotypically, NK cells belong to cytotoxic lymphocytes expressing CD56 and CD16 surface proteins, capable of killing cancer and virus-infected cells without prior immunization. Two populations of NK cells have been distinguished based on the level of CD56 and CD16 expressions: CD56^dim^ CD16^bright^ (high expression of CD16 and strong cytotoxic properties) and CD56^bright^ CD16^dim^ (low expression of CD16 and significant immunoregulatory properties). However, NK cells do not express CD3, which is specific for T lymphocytes [2]. NK cells constitute approximately 10% of lymphocytes circulating in peripheral blood and 90% of this fraction consists of CD56^dim^ CD16^bright^ cells. NK cells originate in the lymphoid lineage of blood cells and participate in innate immune mechanisms [3].

**Figure 1:**
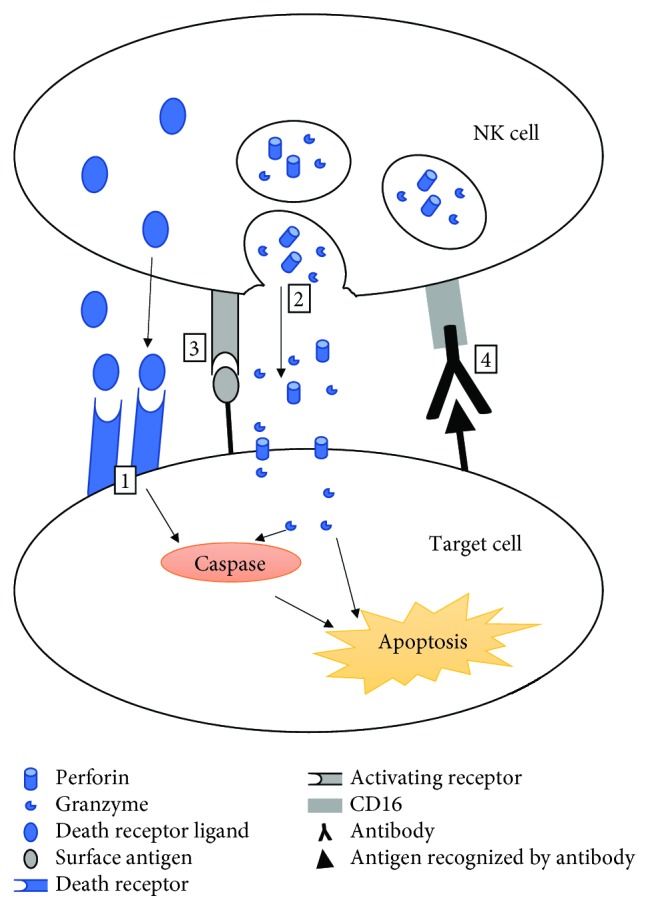
Target recognition and cytotoxicity mechanisms in NK cells. Cytotoxicity mechanisms are colored in blue; target recognition pathways are colored in grey. 1: death receptor pathway, 2: exocytosis of cytotoxic granules, 3: ligand recognition by activating receptor, and 4: antibody-dependent cellular cytotoxicity (ADCC).

NK cells exhibit cytotoxic effects due to direct or indirect target recognition. In the direct pathway, identification occurs through a general signal from NK cell surface receptors that receive activating and inhibiting environmental signals. Molecules recognized by NK cells can be surface glycoproteins present on all nucleated cells, including major histocompatibility complex I (MHC I) or viral antigens. The expression of ligands for activating NK cell receptors must exceed the expression of molecules binding to inhibitory receptors to accomplish target cell lysis. An indirect recognition mechanism called ADCC (antibody-dependent cellular cytotoxicity) utilizes the ability to express the Fc*γ*RIIIa receptor (CD16) by NK cells, which in turn enables antibody-coated target cell detection [4]. The effect of correct target recognition is the activation of killing mechanisms in NK cells: exocytosis of cytotoxic granules or death receptor-mediated cytotoxicity. Cytotoxic granules contain perforin, pore-forming protein, and granzymes—serine proteases. Perforin generates pores in the target cell membrane allowing granzymes to enter the cell and initiate the apoptosis by caspase-dependent and independent pathways [5]. In death receptor-mediated cytotoxicity, death ligands produced by NK cells attach to the death receptors expressed on the target cell surface, thereby activating the caspase cascade leading to the death of the recognized object. Fas ligand, tumor necrosis factor (TNF), TNF-related apoptosis-inducing ligand (TRAIL), and lymphotoxin alpha (LT*α*) have been identified as death receptor ligands [6]. Moreover, NK cells secrete interferon gamma (IFN-*γ*), which regulates various aspects of immune system responses, including NK cell actions, by forming a positive feedback loop [7]. Target recognition pathways and cytotoxicity mechanisms of NK cells are depicted in Figure 1.

Discrimination between healthy and malignant cells by NK cells is crucial; thus, a mechanism called “missing-self recognition” has been proposed. Activating or inhibitory signals are generated during MHC I recognition by NK cells. The ligand indicating the normal cell is recognized by the inhibitory receptor that transmits inhibitory signals and lysis of the target cell does not occur. However, in some disease processes, e.g., tumor development or viral infection, MHC I is often downregulated; thus, the NK cell does not receive inhibitory signals and attacks the target cell [8].

In addition to successful target recognition, activation of NK cell cytotoxicity can also be triggered or enhanced by other factors, either physiological or exogenous. Interleukins (mostly IL-2, IL-12, IL-15, IL-18, and IL-21) play the main role in NK cell activity modulation; their effects were reviewed by many research groups [9–14]; therefore, this topic will not be explained further in this publication. The application of ionophore ionomycin (ION) and phorbol 12-myristate 13-acetate (PMA), which leads to increased NK cell degranulation, is another classical approach used to induce NK cell activity [15]. Apart from the compounds mentioned above, several vitamins and a variety of phytochemicals have been identified as NK cell stimulators. Stimulation effects generally include increased killing of target cells, enhanced IFN-*γ* and TNF*α* production, or higher level of degranulation. Many compounds have also been identified as activators of protein kinase C (PKC), which plays an important role in the lytic signaling pathway in NK cells; hence, its activation is crucial to maintain NK cell cytotoxicity [16].

The aim of the following overview is to present and describe the effects of selected, less-known, NK cell-activating compounds of natural origin. In addition to NK stimulatory effect, the compounds also display tumor-preventing or immunoregulatory properties, making them good candidates for anticancer drugs with a possible wide range of therapeutic applications. This review focuses mostly on describing the role of stimulated NK cells in cancer treatment according to their primary role in the body; however, an additional applications of natural compounds in the other disease aspect are also mentioned. Currently, there are no publications reviewing the list of natural compounds acting as NK cell stimulators; therefore, we hope that this review will help to fill this gap in the field.

## 2. Vitamins

### 2.1. Vitamin A

The term vitamin A includes several groups of fat-soluble compounds, including retinol, retinal, and retinoic acid (RA) along with carotenoids that serve as vitamin A precursors. The idea to investigate the influence of retinoids on NK cells came from the observation that this compound group was able to decrease tumor growth and development in several models. Fraker and colleagues published in 1986 the results of a study conducted on wild-type and athymic BALB/c mice injected with human breast cancer. Administration of retinol increased splenic NK cell activity in wild-type BALB/c mice compared to untreated animals. The highest NK cell activity was obtained 1 h after the treatment [17]. Subsequently, the role of vitamin A in the regulation of NK cell activity was explored using retinol-depleted rats. The activity of splenic NK cells against YAC-1 target cells was lower in vitamin A-depleted rats. The level of IFN-*γ* was measured in order to explain the mechanism of vitamin A action. Samples collected from vitamin A-depleted animals showed reduced IFN-*γ* production, indicating that the lack of vitamin A in rats altered NK cell functioning [18]. In addition to retinol, the effect of retinoic acid and *β*-carotene on NK cell activation was also investigated. A study conducted in 1997 showed that NK cells of athymic mice treated with *β*-carotene showed higher cytolytic activity against YAC-1 cells [19]. Surprisingly, experiments conducted *in vitro* using chicken NK cells treated with retinol resulted in a decreased NK cell activity against LSCC-RP9 target cells; however, observations regarding retinoic acid and *β*-carotene revealed increased NK cell activity [20].

Further investigations performed by Nomura and colleagues in a mice model more precisely explained the connection between retinoic acid and NK cells. Retinoic acid treatment increased the expression of RAE-1 (retinoic acid early inducible gene), identified for the first time in 1994 [21]. The product of RAE-1 is a ligand of the NKG2D receptor located on the surface of NK cells. The activation of NK cells occurs after ligand binding to the receptor [22]. Those results were confirmed in 2007 using hepatic stellate cells as a target. Treatment with retinoic acid sensitized target cells to NK killing by increasing the expression of RAE-1 [23]. Introduction of 13 cis retinoic acid (13cRA) to cancer medicine encouraged researchers to investigate its influence on NK cells during tumor therapy. Clinical trials conducted on metastatic colorectal cancer patients showed that 13cRA in combination with IL-2 displayed a low toxicity profile and increased lymphocyte and NK cell counts [24]. A combination of IL-2, 13cRA, granulocyte-macrophage colony-stimulating factor (GM-CSF), and anti-GD2 antibody was approved as a therapy for neuroblastoma in 2010; however, no data about the influence of this treatment on patients' NK cells were presented [25].

Additionally, a review by Yosaee and colleagues indicates the role of vitamin A in prevention of type 1 diabetes (T1D). Briefly, data from animal studies shows that NK cells are important in the development of T1D; therefore, an increase in NK cell activity caused by vitamin A treatment could be beneficial in T1D treatment [26]. Another review by Oliveira et al. summarizes a bidirectional effect of retinoic acid on NK cells in the context of inflammatory diseases [27].

### 2.2. Vitamin B

Compounds belonging to that class are water-soluble and play crucial functions in cell metabolism. Eight members belonging to the vitamin B group have been identified: B_1_ (thiamine), B_2_ (riboflavin), B_3_ (niacin and nicotinamide riboside), B_5_ (pantothenic acid), B_6_ (pyridoxine), B_7_ (biotin), B_9_ (folate), and B_12_ (various cobalamines). To the best of our knowledge, the relationship between NK cells and vitamins B_1_, B_2_, and B_5_ has not been evaluated; therefore, only findings regarding the remaining compounds are described here.

A study performed by Mamcarz with collaborators and Peng et al. showed that nicotinamide increased the surface expression of CD62L in NK cells. CD62L (L-selectin) receptors participate in NK cell recruitment to the bone marrow and lymph nodes from the circulation; therefore, it is crucial to maintain the expression of this receptor [28, 29]. The depletion of vitamin B_6_, despite its clear association with reduced T cell cytotoxicity, had no effect on NK cell activity in mice [30]. Studies on the relationship between biotin and NK cell activity conducted in patients suffering from Crohn's disease showed that NK cell cytotoxicity was significantly lower in the state of biotin deficiency [31, 32]. Considering the significance of folate in cancer prevention, the influence of vitamin B_9_ was also evaluated in the context of immunology. Trials conducted in women with a folate-deficient diet demonstrated that the presence of unmetabolized folic acid in the serum correlated with lower NK cell toxicity [33]; however, the results from 2013 showed that high doses of folic acid did not change the activity of NK cells *in vitro* [34], suggesting an indirect effect of folic acid on NK cell activity.

Vitamin B_12_ (cobalamines) also participates in immunomodulation. A study in Japanese anemia patients and healthy donors revealed that vitamin B_12_ treatment improved NK cell cytotoxicity in these patients [35]. Moreover, during research performed on aged rats, animals receiving diet deficient in vitamin B_12_ displayed a significant decrease in the activity of splenic NK cells [36]. Another study performed by Erkurt and collaborators shows the influence of cyanocobalamin on immunity in patients with pernicious anemia. The results suggest that vitamin B_12_ displays crucial immunomodulatory effects in the patients, by restoring NK cell activity and normal CD4/CD8 ratio [37].

Despite the promising interactions between various forms of vitamin B and NK cells, the mechanism of vitamin B actions remains elusive and requires supplementary research.

### 2.3. Vitamin C

Vitamin C (ascorbic acid; AA) is water-soluble; it serves as an antioxidant agent and participates in many enzymatic reactions, e.g., wound healing. In contrast to vitamin B, the effects of AA were investigated in more details. Initial trials started in 1980s, when Huwyler's group measured NK cell activity after AA treatment of lymphocytes isolated from human peripheral blood. The results indicated the inhibitory function of AA on human NK cell activity in a dose-dependent manner after 1 h of incubation with the compound. Furthermore, NK cell activity induced by IFN-*γ*, IL-2, or effector-target binding was not disrupted by the addition of AA [38]. The results obtained in a toad model (*Bufo marinus*) also showed a decrease in NK cell activity. *In vitro* tests conducted in peripheral blood lymphocytes (PBL) treated with AA showed that the compound did not change the viability of these cells; however, a significant inhibition of toxicity against YAC-1 cells was evident. *In vivo* trials also demonstrated a decrease in NK cell toxicity in a time-dependent manner [39]. In contrast, a study conducted by Vojdani and Ghoneum showed increased NK cell activity in healthy individuals 8 hours after oral AA administration. This effect was also visible after 24 hours, and then the activity returned to its normal level after 48 hours [40]. Next, Sefton and Taddie attempted in 1997 to explain the mechanism of AA action on NK cells. The aim of this study was to analyze the effect of vitamin C in patients previously exposed to toxic chemicals. Investigated blood samples included the material collected before and 24 hours after vitamin C administration. The discovery of protein kinase C (PKC) in lymphocyte metabolism [41] encouraged the researchers to investigate PKC involvement in NK cells in response to vitamin C treatment. Again, the activity of NK cells was higher after AA administration. Moreover, high PKC levels measured by ELISA correlated with higher NK cell toxicity, suggesting that the activation of the PKC signal transduction pathway was responsible for NK cell activation [42]. The findings regarding NK cell activation by AA were also supported by more recent data published by Huijskens and colleagues. These researchers confirmed the rise in NK cell cytotoxicity in *in vitro* assay using peripheral blood mononuclear cells incubated with vitamin C. Moreover, AA appears to stimulate the differentiation of NK cell progenitors from hematopoietic stem cells and T/NK cell progenitors, suggesting a role of this compound in NK cell maturation [43]. Except cancer treatment, the influence of vitamin C on NK cells was also investigated in the context of influenza A virus/H1N1 infection. A research conducted by Kim with colleagues showed that the administration of vitamin C and red ginseng synergistically increased the expression of NKp46, CD69, and CD25 and IFN-*γ* production in NK cells indicating a rise in NK cell activity [44]. Nevertheless, more research is required to fully discover the potential of vitamin C in NK cell biology.

### 2.4. Vitamin D

Vitamin D belongs to steroid compounds insoluble in water. In humans, 1,25-dihydroxyvitamin D (1,25D) is the most active form of vitamin D. Deficiencies of 1,25D in humans may lead to cancer development, rickets, disturbed bone mineralization, and immune responses. A study published in 1999 evaluated the influence of 1,25D on NK cells with a particular emphasis on PKC involvement in this process. The established YT NK cell line was used as a model. RT-PCR and Western blot showed an increase in *α*, *β*, *δ*, *ε*, *ζ*, and *θ* and a decrease in *η* and *μ* PKC isoforms after 1,25D treatment. Moreover, the level of BLT-esterase (a marker of NK lytic granules) was significantly higher in 1,25D-treated samples [45]. Subsequent studies performed in NK cells isolated from blood donated by healthy volunteers showed that 1,25D and its synthetic analog, calcipotriol increased the lysis of K562 and RAJI cells by NK cells preactivated with IL-2. Additionally, both treatments caused a rise in the expression of NK-activating receptors: NKp30, NKp44, and NKG2D and downregulation of the inhibitory receptor, CD158. Moreover, the population of NK cells—NK17/NK1—also exhibited higher cytotoxic activity against K562 cells after 1,25D and calcipotriol treatment [46]. In contrast, studies conducted in 1989 and 2015 presented data supporting the inhibitory effect of 1,25D on NK cells. 1,25D inhibited NK cell toxicity and decreased the activity of preactivated NK cells with the use of IL-2 and IFN-*γ* [47]. Furthermore, 1,25D decreased the expression of the activating receptor CD69 and upregulated the expression of CD158a and CD158b. Moreover, secretion of IFN-*γ* and TNF*α* was lower in treated NK cells [48]. The importance of the appropriate levels of vitamin D was also investigated in rheumatoid arthritis development [49]; however, the data regarding the influence of 1,25D on NK cells in autoimmune diseases is not consistent (reviewed by Dankers et al. [50]).

The connection between 1,25D and NK cells certainly requires further research, since currently available data are not consistent and do not allow to formulate clear conclusions.

### 2.5. Vitamin E

The term vitamin E refers to a group of water insoluble compounds that include tocopherols and tocotrienols. These substances act as antioxidants, and therefore, they effectively disrupt the generation of reactive oxygen species, which is important in the anticancer response. The idea that led to the study, published in 1999, was based on the fact that the activity of NK cells declined during ageing and it could be possible to restore NK cell cytotoxicity. Among several investigated antioxidants, *α*-tocopherol was used. The data from an aging mice study showed that all of the investigated compounds were able to enhance NK cell activity [51]. Another work also conducted in mice that were supplemented with *α*-tocopherol resulted in an increased NK cell activity, and *in vitro* treatment of NK cells showed elevated levels of tumor-lytic activity of these cells [52]. Similarly, short dietary intake of higher vitamin E doses improved lytic activity of NK cells in patients suffering from colorectal cancer [53]. Additionally, diet rich in vitamin E and selenium had a positive effect on NK cell cytolytic function in cattle (Nellore bulls) [54]. Moreover, the effect of vitamin E treatment was investigated in mice suffering from AIDS. Vitamin E administration increased NK cell toxicity and IFN-*γ* levels which were suppressed by retrovirus infection [55]. Although data regarding the influence of *α*-tocopherol on NK cells are consistent, indicating the stimulatory effect of vitamin E on NK cells, no mechanism of action at the molecular level has been proposed yet. Considering the effect of various antioxidants in cancer treatment and prevention, further investigations involving molecular mechanisms of NK cell activation with these drugs may be beneficial.

## 3. Phytochemicals

### 3.1. Genistein

Genistein is an isoflavone compound found in soybean. It not only acts as an antioxidant but also mimics estrogen hormone by binding to its receptor. The idea to investigate the effect of genistein on NK cell activity came from the observation that the rate of prostate, breast, and colon cancer was smaller in the countries with higher dietary soybean intake [56].

A study performed by Zhang and colleagues showed that genistein in the concentration range of 0.1-0.5 *μ*mol/l increased NK cell activity; however, this activity declined with increasing doses at concentrations above 0.5 *μ*mol/l. Moreover, genistein metabolite—genistein glucuronide—showed a similar effect as genistein, although it required a broader range of concentrations [57]. Subsequent studies evaluated the influence of different genistein contents in the rat diet. As a result, the number of splenic NK cells decreased in all three investigated groups with low (L), medium (M), and high (H) genistein administration; however, there was no effect on NK cell cytotoxicity in the F1 generation. The effect was different in the F0 generation, which showed increased NK cell activity in groups M and H. The authors proposed a hypothesis that genistein could have a biphasic effect on NK cells [58]. Guo with colleagues investigated further the impact of genistein on NK cells, resulting in the confirmation of stimulatory effect on NK cell triggered by genistein [59]. Additionally, a research conducted of mice model of upper airway inflammation showed that after isoflavone treatment, NK cell degranulation was increased [60].

### 3.2. Curcumin

Curcumin is an active component of *Curcuma longa* and exerts a wide spectrum of desired biological effects, i.e., it has anti-inflammatory effect, acts as an antioxidant, reduces cholesterol levels, and modulates histone activity. Currently, it is investigated worldwide as an anticancer agent. Experiments conducted on curcumin-treated rats showed that the increased dose of curcumin resulted in elevated NO production by NK cells, thereby causing apoptosis in target cancer cells. In addition, cytotoxicity against AK-5 and YAC-1 target cells was higher after the treatment [61]. Similarly, research conducted on NK cells against K562 target cells resulted in higher NK cell toxicity in treated samples [62]. Moreover, curcumin-pretreated breast tumor cells secreted exosomes that exerted lower inhibitory effect on NK cell activity than exosomes from nontreated tumors [63]. The review published by Fiala well explains the action of curcumin combined with omega-3 fatty acid. The data gathered by the latter author support the thesis that curcumin in combination with omega-3 increases NK cell-induced apoptosis of pancreatic cancer by inhibiting NF-*κ*B; however, IFN-*γ* production is suppressed, which can cause some undesired effects in the context of cancer therapy [64]. Certainly, further *in vivo* research is required to better understand the benefits and disadvantages of cancer therapy using curcumin; however, the results from phases I and II of clinical trials obtained so far have suggested that curcumin tolerance in patients is high when administered orally at doses up to 12,000 mg/day [65, 66]. An importance of curcumin in other types of diseases with an indication of the role of curcumin in NK cell activation was summarized in a review by Jagetia and Aggarwal [67]. Briefly, curcumin acts like an immunomodulatory agent and also supports cancer chemotherapy and therefore can serve as a potential drug for arthritis, diabetes, asthma, psoriasis, and several other diseases.

### 3.3. Ginseng Extract

Ginseng is a plant species widely use in Chinese medicine due to its immunomodulatory and antioxidant effects. In 1987, Yun and colleagues tested the influence of red ginseng on NK cell activity in mice with lung carcinoma. Decreased NK cell activity caused by chemical mutagens was restored by red ginseng treatment, suggesting a relationship between the compound and the killer cell population [68]. Subsequently, a study on methanol extract of ginseng cambial meristematic cells (MEGCs) revealed elevated activity of NK cells, measured by the 4h-^51^Cr release assay. Moreover, increased granzyme B expression was present in MEGC-treated cells when compared to control. However, no significant changes occurred in the NK cell count and expression of their activating and inhibitory receptors [69]. In another analysis, the aqueous ginseng extract was orally administered to mice, and then tumor-lytic activity of NK cells against YAC-1 cells was measured. Those experiments revealed that NK cells were activated by aqueous ginseng extract in an IFN-*γ*-dependent manner [70]. Furthermore, studies conducted by See and colleagues revealed that extracts from ginseng enhanced NK cell functions both in healthy individuals and patients suffering from chronic fatigue syndrome or acquired immunodeficiency syndrome [71].

### 3.4. Garlic Extract

The garlic species belongs to the genus *Allium* and is used worldwide as an intense flavoring. Additionally, it has been used for centuries as a natural remedy, and for this reason, several studies focused on revealing the potential connection between garlic extracts and NK cell activity.

The experiments performed by Hassan and colleagues using fresh garlic extract showed that this substance applied to tumor-bearing mice caused an increase in NK cell activity in these animals. Further studies on the extract allowed to identify a fraction with possible stimulatory effect. The R10 fraction, injected daily to mice, significantly reduced tumor growth [72]. In addition, research conducted on patients suffering from liver, pancreatic, or colon cancer, who were treated with aged garlic extract (AGE), demonstrated a significant increase of both NK cell number and NK cell activity in the AGE group; however, the quality of patients' life did not improve [73]. The following clinical trials conducted by Xu and collaborators investigated the effect of AGE supplementation in healthy obese adults. Although there was no difference in NK cell percentage between treated and nontreated groups, AGE administration modified the inflammation and immunity of the adults with obesity [74]. The results of garlic extract administration seem promising, but the identification of the active compound in the extract is crucial for further investigating this topic in the context of immunity.

### 3.5. Resveratrol

Resveratrol is a phenolic compound with antioxidant properties, found in the skin of certain fruits, such as grapes, blueberries, or raspberries. Studies regarding the role of resveratrol in cancer treatment were extensively reviewed by Rauf and colleagues [75]. An investigation performed on rats conducted by Lu and coworkers showed that resveratrol pretreatment for 3 days significantly increased NK cell activity measured in the ^51^Cr release assay [76]. Subsequently, the group conducted another study aiming at resolving the molecular mechanism of resveratrol effect on NK cells. The results using the NK92 cell line as a model of NK cells showed that resveratrol increased the toxicity of effectors against K562, HepG2, and A549 cell lines in a dose-dependent manner. The effect was correlated with increased JNK and ERK1/2 MAP kinase activity and perforin expression. Additionally, this treatment upregulated the expression of the NKG2D receptor. In addition, the possible additive effect of resveratrol with IL-2 was postulated, although the reported results did not confirm this type of reaction. The authors concluded that resveratrol in NK92 cells can act via similar or overlapping pathway as IL-2 in the context of increased NK cell cytotoxicity [77]. Those results were supported by Chinese researchers, who also reported the stimulatory effect of resveratrol on INF-*γ* secretion [78].

### 3.6. Ashwagandha Extract

Ashwagandha, also known as *Withania somnifera*, is a plant that belongs to the family *Solanaceae* and is used in Ayurveda medicine. Ashwagandha was successfully applied in studies on rat models of arthritis [79] and in trials involving schizophrenia patients [80]. Moreover, ashwagandha extracts were also responsible for the activation of NK cells. Mikolai and colleagues investigated the effect of ashwagandha extracts administered with milk to healthy participants on their immune cells. The group discovered a significant increase in NK cell activity after 96 hours, indicated by higher CD69 expression [81]. Similar results were obtained by Malik and coworkers in their trials on tumor-bearing mice. Animals treated with the plant extract displayed an increase in NK cell population in blood samples stained with anti-mouse NK1.1 antibody [82]. Another study conducted in hens susceptible to ovarian cancer development showed that ashwagandha supplementation reduced the incidence of tumor development; changes in NK cell population were also observed. Both stromal and intratumoral NK cell populations significantly increased after supplementation, suggesting a protective function of NK cells against carcinogenesis [83]. The influence of ashwagandha extracts on NK cells requires both the identification of an active compound in the extracts and further studies on its mechanism of action. Considering such promising results in the context of cancer research, this area is worth deeper investigation.

### 3.7. Ingenol Mebutate

Ingenol mebutate (IM), also known as ingenol-3-angelate, PEP005, or the commercially available Picato, is a compound isolated from the plant *Euphorbia peplus*. In medicine, Picato is used in topical treatment of actinic keratosis [84, 85]. A study aiming to determine the influence of IM on PKC activity showed that IM could act as a PKC ligand with a high binding affinity to PKC*α* and *δ* isoforms [86]. The impact of IM on NK cells is not yet well investigated. To the best of our knowledge, only one research group attempted to publish the results regarding such an interaction. In 2016, Garrido with colleagues investigated the outcome of IM treatment on antiviral activity, cytotoxicity, cytokine secretion, and viability of NK cells. The results indicated that IM impaired NK cell viral activity. Moreover, degranulation of NK cells, measured by CD107a expression, was also impaired after IM treatment [87]. Considering the high toxicity of IM, but also a stimulatory effect on PKC activity in NK cells, it is worth to reconsider the idea of using IM as NK cell activator in cancer treatment. This compound could help, for instance, in designing IM analogs with lower cytotoxicity, but the same or greater effect on PKC.

### 3.8. Kumquat Pericarp Extract

Kumquat (*Fortunella crassifolia*) is a small, orange fruit belonging to the genus *Citrus*. In addition to its pleasant taste values, kumquat is also considered to have some beneficial health-promoting effects, for example, antimicrobial activity [88] and antioxidant properties [89]. In 2015, Nagahama and colleagues published the first attempt to characterize the influence of kumquat on NK cell activity *in vitro* and *in vivo*. Kumquat pericarp acetone fraction (KPAF) was used as a stimulant in the latter study. An *in vitro* study conducted on the KHYG-1 NK cell line showed that IFN-*γ* production and NK cell cytotoxic activity against K562 and YAC-1 cells were significantly higher after treatment. Subsequent mouse experiments also resulted in elevated NK cell toxicity and IFN-*γ* production. Several predicted constituents were tested as stimulators of IFN-*γ* expression in KHYG-1 cells to identify the active compound in KPAF. *β*-Cryptoxanthin was the only substance among tested compounds that demonstrated the stimulatory effect, indicating that the mechanism of NK cell activation by KPAF may be caused by carotenoids [90]. At present, no other data regarding kumquat effect on NK cells are available; thus, this area requires more studies explaining the role of kumquat in immunology and cancer research.

### 3.9. Prostratin

Prostratin, similarly to IM, belongs to PKC agonists and was also isolated from a plant species *Homalanthus nutans* belonging to the *Euphorbiaceae* family. Chemically, prostratin is a phorbol ester, but in contrast to PMA, it is not carcinogenic [91]. The study of Garrido research group, mentioned in the IM section, also included the effect of prostratin on NK cell activity. Prostratin improved significantly NK cell antiviral activity, but impaired killer degranulation and cytokine production compared to untreated cells. Prostratin also increased the expression of the activating receptor NKG2D [87]. In conclusion, only one available study using prostratin as an NK cell stimulator is not sufficient to develop a cancer treatment strategy, but it is a good start to further examine the effects of this PKC activator on NK cells and its possible benefits in cancer treatment.

### 3.10. Lectins

Lectins belong to the group of proteins able to recognize and bind saccharide structures non-covalently and reversibly. In cancer research, mistletoe (*Viscum album L.*) extracts containing lectins were widely investigated. A review by Braedel-Ruoff summarizes the results of clinical trials checking the influence of mistletoe extract Iscador® on NK cells [92]. Briefly, several studies showed a positive effect of mistletoe extracts on NK cells; however, some future studies are still required.

### 3.11. Polysaccharides

Numerous studies regarding the impact of different polysaccharides on NK cells were conducted; therefore, this publication mentions only a few most recent findings. A study by Surayot and You describes the effects of sulfated polysaccharides (SP) from seaweed *Codium fragile.* SP increased the proliferation of NK cells and their toxicity against HeLa cells. Moreover, the expression of NKp30 receptor and the secretion of IFN-*γ* and perforin with granzyme B were also elevated; however, more research is required to know the exact mechanism of SP influence on NK cells [93]. Another research focused on the antitumor activities of polysaccharides isolated from green tea leaves (GTLP). The authors discovered that GTLP were able to increase NK cell toxicity against YAC-1 cells and activate macrophages [94]. Finally, one study using polysaccharides extracted from animal origin (oyster) resulted in stimulation of the activity of mouse spleen NK cells *in vitro* and *in vivo* [95]. In conclusion, polysaccharides have also a stimulating effect on NK cells and might be an additional source of compounds supporting cancer treatment.

## 4. Summary and Conclusions

The approach to battle cancer using immunology mechanisms attracts more researchers' attention every year. Therapies based on NK cell utilization encourage searching new compounds with stimulatory properties and minimal side effects and simultaneous strong influence on lymphocyte activation. Most of the studies cited in this review first focused on analyzing the general effect of the investigated compound on NK cell activity and then tried to explain the mechanism behind the desired result. Although the effect of treatment on tumor development is crucial, the molecular mechanism is also important, because better understanding of the process can lead to designing therapies with improved efficacy and lower side effects. Furthermore, not only the influence of the compound on lymphocytes should be determined but also the effect on cancer cells and tumor microenvironment. The stimulatory effect is sometimes caused by a mixture of active compounds isolated from the plant; therefore, identification of composites with favored properties should be a priority; however, purification of a particular group of molecules can be difficult. Knowledge about the structures can also be useful in designing analogs. Modern chemistry allows to modify basic compounds on multiple levels, thereby obtaining derivatives with improved properties.

**Figure 2:**
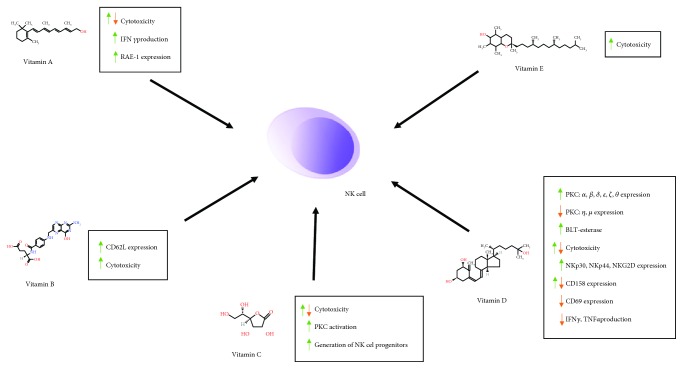
Effects of vitamins A, B, C, D, and E (schematically represented by a chosen member from each group) on NK cell activity.

Although the data gathered above and summarized in Figure 2 and Table 1 do not allow selecting one, the best and safest compound as a NK cell stimulator, the results regarding plant extracts seem promising and are worth further investigations. The exact mechanisms of vitamin actions, either alone or in combinations, should also be carefully determined due to their wide availability and role in cancer treatment. Finally, a variety of compounds with established PKC stimulatory activity should be tested as NK cell stimulators, alone or in combinations with other natural or synthetic substances.

## Figures and Tables

**Table 1:** The effect of natural compounds on NK cells.

Substance name	Effect on NK cells	References
Genistein	Increased/decreased cytotoxicity	[56–60]
Curcumin	Increased NO production, increased cytotoxicity	[61–67]
Ginseng extract	Increased cytotoxicity and granzyme B expression	[68–71]
Garlic extract	Increased cytotoxicity and cell number	[72–74]
Resveratrol	Increased cytotoxicity, JNK, ERK1/2 MAP kinase activity, perforin and NKG2D expression, and IFN-*γ* production	[75–78]
Ashwagandha extract	Increased cell number and CD69 expression	[79–83]
Ingenol mebutate	PKC activation, impaired cytotoxicity, and degranulation	[84–87]
Kumquat pericarp extract	Increased cytotoxicity and IFN-*γ* production	[88–90]
Prostratin	PKC activation, increased NKG2D expression and antiviral activity, impaired degranulation, and cytokine production	[87, 91]
Lectins	Increased cytotoxicity	[92]
Polysaccharides	Increased cytotoxicity and proliferation	[93–95]
